# Vascular access type and prognosis in elderly hemodialysis patients: a propensity-score-matched study

**DOI:** 10.1080/0886022X.2024.2387205

**Published:** 2024-08-09

**Authors:** Ru-xin Liu, Shuai Lin, Li Liu, Juan Xu, Lin-na Liu, Jie Pang, Hai-wen An, Wen-qin Yang, Jian-lin Jian, Jin Wang, Zhi-lan He, Xiao-lan Luo, Hui Zou, Yuan Zeng, Qing-xiu Huang, Yan-lin Li

**Affiliations:** Department of Nephrology, Zhongshan Hospital of Traditional Chinese Medicine Affiliated to Guangzhou University of Traditional Chinese Medicine, Zhongshan, China

**Keywords:** Hemodialysis, arteriovenous fistula, tunneled cuffed catheter, mortality, propensity score matching

## Abstract

**Background:**

To compare the impact of tunneled cuffed catheters (TCCs) and arteriovenous fistulas (AVFs) on outcomes in elderly hemodialysis (HD) patients.

**Methods:**

A retrospective matched cohort study was performed. Propensity score matching (PSM) was applied to balance the baseline conditions, and we compared all-cause mortality, major adverse cardiovascular and cerebrovascular events (MACCEs), hospitalization, and infection rates between AVF and TCC patients ≥70 years old. Cox survival analysis was used to analyze the risk factors for death.

**Results:**

There were 2119 patients from our center in the Chinese National Renal Data System (CNRDS) between 1 January 2010 and 10 October 2023. Among these patients, 77 TCC patients were matched with 77 AVF patients. There was no significant difference in all-cause mortality between the TCC and AVF groups (30.1/100 *vs.* 33.3/100 patient-years, *p* = 0.124). Among the propensity score-matched cohorts, no significant differences in Kaplan–Meier curves were observed between the two groups (log-rank *p* = 0.242). The TCC group had higher rates of MACCEs, hospitalization, and infection than the AVF group (33.7/100 *vs.* 29.5/100 patient-years, 101.2/100 *vs.* 79.5/100 patient-years, and 30.1/100 *vs.* 14.1/100 patient-years, respectively). Multivariate analysis showed that high Charlson comorbidity index (CCI) score was a risk factor for death.

**Conclusions:**

There was no significant difference in all-cause mortality between elderly HD patients receiving TCCs and AVFs. Compared with those with a TCC, elderly HD patients with an AVF have a lower risk of MACCEs, hospitalization, and infection.

## 1. Introduction

The global burden of end-stage renal disease (ESRD) is rapidly increasing, with a growing number of elderly patients requiring renal replacement therapy [1]. This rise is further fueled by a global aging population, leading to a larger proportion of elderly patients requiring hemodialysis. In the United States, 52% of hemodialysis patients are 65 years or older [2]. Hemodialysis (HD), a renal replacement therapy for ESRD, relies on establishing a reliable vascular access (VA) for blood flow during the procedure. There are three main types of VA: arteriovenous fistula (AVF), arteriovenous graft (AVG), and central venous catheter (CVC), further research is needed to determine the optimal access method specifically for elderly patients [3].

According to previous guidelines, the ‘fistula first’ concept has been advocated for initial hemodialysis patients [4]. Many studies have shown that patients with an AVF have a greater survival rate than patients with other vascular access types, while patients with a CVC have a higher mortality rate [5–9]. However, AVF requires higher demanding vascular conditions. It should be considered whether elderly HD patients who are in poor vascular condition, have comorbidities, or have a relatively short life expectancy can derive the same benefits from AVF because of the lower rate of AVF maturation [10,11]. The ‘Patients first’ concept is recommended in the 2019 KDOQI VA guidelines, which emphasizes that clinicians should choose vascular access for hemodialysis according to the patient’s actual situation, such as the patient’s current medical condition, life expectancy, and preference [2].

Current clinical guidelines and recommendations provide little clear information on how to select the type of vascular access for elderly patients. The selection of the vascular access type for elderly HD patients is a complex problem. More clinical trials are needed to verify which vascular access is suitable for elderly HD patients. Therefore, we conducted a study in our center to evaluate the effect of tunneled cuffed catheters (TCCs) and arteriovenous fistulas (AVFs) on the prognosis of HD patients ≥70 years old by using a propensity-score-matched cohort.

## 2. Materials and methods

### 2.1. Study cohort

The study was approved by the institutional review board of Zhongshan Hospital of Traditional Chinese Medicine Affiliated to Guangzhou University of Traditional Chinese Medicine (approval No. 2024ZSZY-LLK-025) and complied with the Declaration of Helsinki. The requirement for informed consent was waived because the study was retrospective. This retrospective cohort study included all patients in the Chinese National Renal Data System (CNRDS) who underwent maintenance dialysis at our center between 1 January 2010 and 10 October 2023. According to the 2019 KDOQI VA guidelines [2], CVCs can be used for long-term and short-term use. In our study, a TCC was defined as a tunneled cuffed catheter that was a long-term CVC. Our center suggests that patients who (1) have multiple prior failed AV accesses with no available options, (2) are absence of AV access creation options due to a combination of inflow artery and outflow vein problems, (3) have more comorbidities, (4) have a limited life expectancy, or (5) refuse AVF placement should attempt at TCC placement for hemodialysis. The inclusion criterion was judged by a clinician. Patients who (1) lacked baseline data, (2) were followed up for <3 months, or (3) were younger than 70 years were excluded. Full-time staff were responsible for the system information registration and all of the follow-up of the maintenance dialysis patients in our center. Hence, the data of the cohort were relatively complete and reliable.

### 2.2. Data collection

Data on baseline demographics, comorbid conditions, and laboratory test results were obtained from our inpatient system and then compared with the data from the CNRDS. The demographic data included birth, sex, initial vascular access (AVF or TCC), date of first dialysis, and primary kidney disease status. Comorbidities were identified at baseline by the International Classification of Diseases, 9th and 10th Revision (ICD-9 and ICD-10) codes in the medical record, and the Charlson comorbidity index (CCI) was calculated based on Quan et al.’s method [12]. The laboratory indicators included blood urea nitrogen, serum creatinine, triglycerides, cholesterol, plasma albumin, and hemoglobin.

### 2.3. Outcomes and exposures

The main outcome was all-cause mortality. The secondary outcomes were main adverse cardiovascular and cerebrovascular events (MACCEs), hospitalization, and infection. In this study, the MACCEs included cerebral hemorrhage, stroke, heart failure, myocardial infarction, unstable angina, peripheral vascular events, and sudden death [13]. All infections included all other infectious diseases except upper respiratory tract infections. Follow-up was continued until one of the following censored events occurred: switched to another dialysis modality, received a kidney transplant, transferred to another dialysis center, death, or reached the end of follow-up (10 October 2023). The outcome data were retrospectively retrieved from the CNRDS and inpatient systems.

### 2.4. Statistical analysis

All the statistical analyses in this study were performed using IBM SPSS (version 25.0) and GraphPad Prism (version 8.0). Normally distributed data are expressed as the mean ± standard deviation (*SD*), and *t*-tests were used for comparisons. Non-normally distributed variables are expressed as the median with range, and the Mann–Whitney *U* test was used for comparisons between the groups. Categorical variables are presented as numbers (percentages) and were analyzed by the chi-square (*χ*^2^) test. The *χ*^2^ test was used to compare the exposure-adjusted rate of patient-level events between the TCC patients and matched AVF patients.

The Kaplan–Meier survival curves were used to compare overall survival and the occurrence of the first MACCE between patients with different vascular access types, and the difference was tested for significance by the log-rank method. Univariate and multivariate Cox proportional hazards regression models were used to compare the hazard ratios (HRs) with 95% confidence intervals (CIs) for death between the TCC and AVF patients, using the time from initial dialysis to censoring as the timescale. Results with a *p*-value <0.05 were considered statistically significant.

Propensity-score-matched analysis reduces bias resulting from the nonrandom nature of the treatment assignment seen in observational studies [14].

## 3. Results

### 3.1. Baseline characteristics

**Figure 1.**
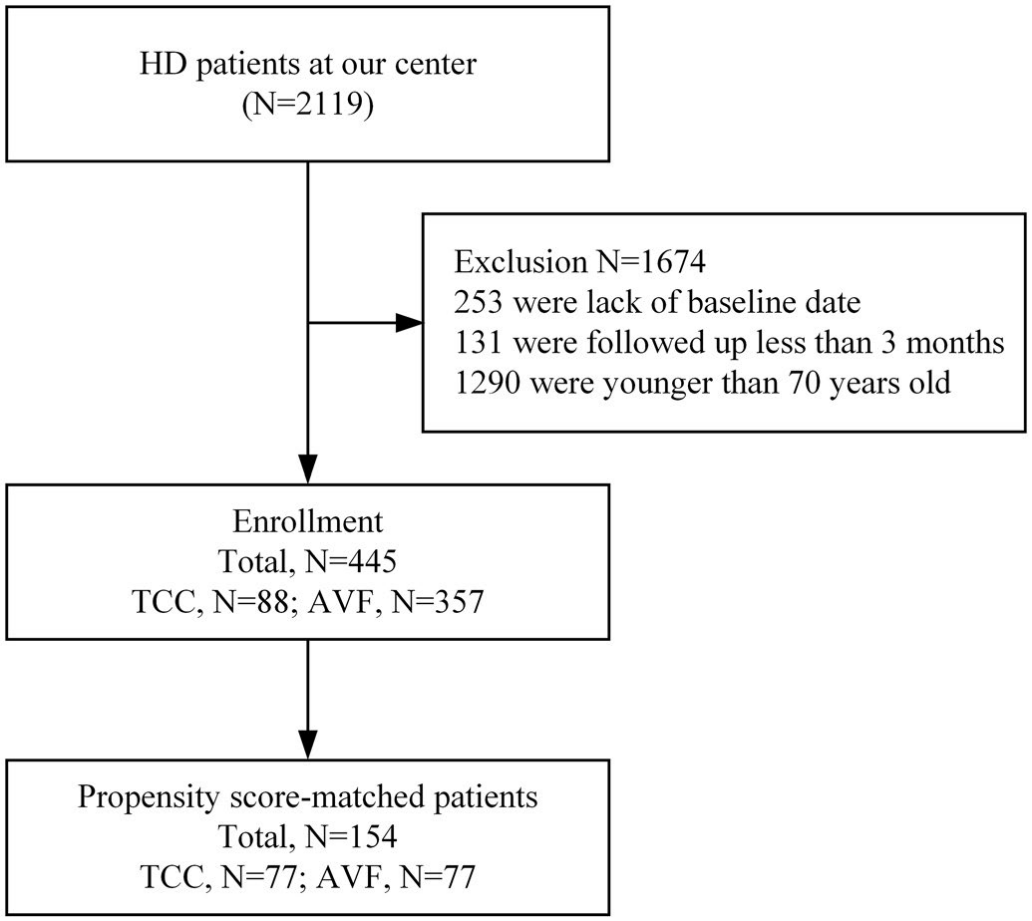
Study profile. HD: hemodialysis; TCC: tunneled cuffed catheter; AVF: arteriovenous fistulas.

The study cohort profile is shown in Figure 1. There were 2119 patients in the CNRDS who were registered by our center during the study period. A total of 1675 patients were excluded for the following reasons: lack of baseline data (*n* = 217), follow-up of <3 months (*n* = 611), and age younger than 70 years (*n* = 847). Among the 445 patients included, 88 patients had a TCC, and 357 patients had an AVF. A propensity score was calculated for this cohort, and patients with TCC were propensity score-matched 1:1 with those for whom AVF was selected. A total of 77 matched pairs of patients were included in the final analyses.

The baseline characteristics of the TCC patients and matched AVF patients are shown in Table 1. Before propensity score matching, the differences in both age and CCI score between the TCC and AVF patients were significant. Compared with AVF patients, TCC patients were older [79 (74, 84) *vs.* 75 (72, 80), *p* < 0.001] and presented a higher CCI [6 (5, 7) *vs.* 6 (6, 8), *p* < 0.001].

**Table 1.** Baseline characteristics of study population at the time of dialysis initiation before and after PSM.

Characteristics	Before matching			After matching		
	TCC (*n* = 88)	AVF (*n* = 357)	*p*	TCC (*n* = 77)	AVF (*n* = 77)	*p*
Gender			0.176			0.519
Female	44	150		36	40	
Male	44	207		41	37	
Age at start of hemodialysis (years)	79 (74, 84)^#^	75 (72, 80)^#^	<0.001	78 ± 5	77 ± 5	0.827
Causes of ESRD (%)			0.058			0.209
Glomerulus nephritis (*n*)	22 (25%)	113 (31.7%)		20 (26%)	27 (35.1%)	
Diabetic nephropathy (*n*)	33 (37.5%)	89 (24.9%)		26 (33.8%)	29 (37.7)	
Other or unknown (*n*)	33 (37.5%)	155 (43.4%)		31 (40.3%)	21 (27.3)	
CCI score^#^ laboratory values	6 (5, 7)	6 (6, 8)	<0.001	6 (5, 7)	6 (5, 7)	0.503
Serum urea (mmol/L)				22.9 (16.5, 31.4)^#^	23.1 (17.4, 31.2)^#^	0.965
Serum creatinine (umol/L)				675 (289, 805)^#^	634 (506, 757)^#^	0.069
Hemoglobin (g/L)				81.6 ± 20.5	85.6 ± 20.8	0.224
Plasma albumin (g/L)				33.6 ± 5.2	34.0 ± 5.8	0.531
Triglyceride (mmol/L)^#^				1.35 (0.99, 1.97)	1.31 (0.99, 1.87)	0.868
Cholesterol (mmol/L)				4.6 ± 1.6	4.3 ± 1.3	0.196

TCC: tunneled cuffed catheter; AVF: arteriovenous fistulas; LDL-C: low-density lipoprotein cholesterol; CCI score: Charlson comorbidity score; IQR: interquartile range; PSM: propensity score matching.

^#^Values are presented as median and interquartile range.

After propensity score matching, there were no significant differences in sex, age, Charlson comorbidity index (CCI) score, cause of end-stage renal disease (ESRD), or biochemical measures between the two groups, which suggested that these patients were likely eligible for either vascular access.

### 3.2. Patient-level outcomes

#### 3.2.1. Mortality

**Figure 2.**
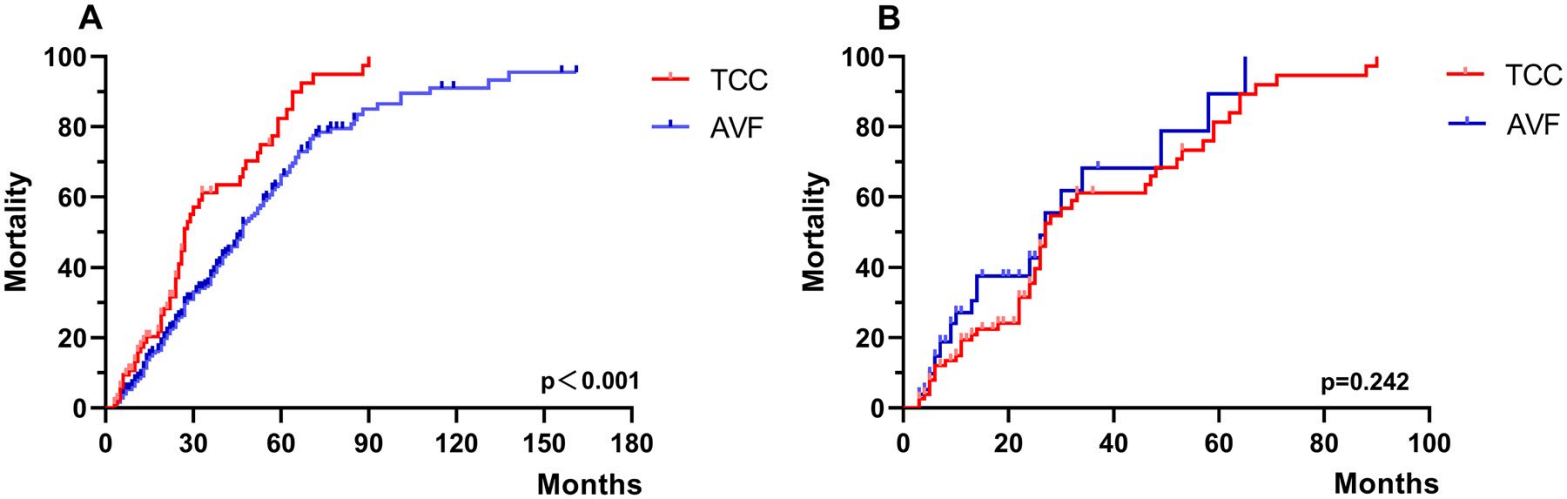
Kaplan–Meier survival for the TCC and AVF patients. (A) Patients before matching; (B) patients after PSM. TCC: tunneled cuffed catheter; AVF: arteriovenous fistulas; PSM: propensity score matching.

The Kaplan–Meier survival curve showed better survival for the AVF patients than for the TCC patients (log-rank *p* < 0.001, Figure 2(A)) before matching. However, the Kaplan–Meier curves were not significantly different between the two groups (log-rank *p* = 0.242, Figure 2(B)) after matching. The median survival time was 27.0 months for the TCC patients and 27.1 months for the AVF patients.

As shown in Table 2, among the propensity score-matched cohorts, the exposure-adjusted mortality was 30.1 per 100 patient-years in the TCC group and 33.3 per 100 patient-years in the matched AVF group (*p* = 0.124). Both univariate and multivariate Cox survival analyses indicated that TCC and AVF were not significantly associated with mortality. Moreover, higher CCI scores were a risk factor (Table 3).

**Table 2.** Patient-level outcomes between TCC and AVF after PSM.

Events	TCC (*n* = 77, 166 patient-years)		AVF (*n* = 77, 78 patient-years)		p*
	No. of events	Exposure-adjusted rate (per 100 patient-year)^#^	No. of events	Exposure-adjusted rate (per 100 patient-year)^#^	
Death	50	30.1	26	33.3	0.124
MACCE	56	33.7	23	29.5	0.043
Hospitalization	168	101.2	62	79.5	<0.001
Infection	50	30.1	11	13.6	<0.001

MACCE: main adverse cardiovascular and cerebrovascular events; Infection: includes all other infectious diseases except the upper respiratory tract infection; TCC: tunneled cuffed catheter; AVF: arteriovenous fistulas; PSM: propensity score matching.

*p-Values were calculated for the exposure-adjusted incidence rate.

^#^The exposure-adjusted rate was calculated as 100 times the total number of events divided by the total number of patient-years of exposure.

**Table 3.** Risk factors for mortality and the occurrence of first MACCE assessed by cox regression model after PSM.

Variables	Death		MACCE	
	Crude HR (95% CI)	Adjusted HR (95% CI)	Crude HR (95% CI)	Adjusted HR (95% CI)
Male	0.981 (0.619, 1.555)	1.106 (0.671, 1.822)	0.878 (0.509, 1.514)	0.856 (0.473, 1.550)
TCC	0.744 (0.452, 1.223)	0.677 (0.390, 1.177)	1.814 (0.983, 3.349)	2.062 (1.081, 3.932)
Age at start of hemodialysis	0.982 (0.928, 1.039)	0.956 (0.898, 1.017)	1.021 (0.959, 1.087)	1.006 (0.942, 1.074)
Cause of ESRD				
Glomerulus nephritis (*n*)	Ref.	Ref.	Ref.	Ref.
Diabetic nephropathy (*n*)	0.689 (0.392, 1.211)	0.644 (0.286, 1.450)	0.948 (0.482, 1.864)	1.140 (0.491, 2.647)
Other or unknown (*n*)	0.612 (0.346, 1.083)	0.564 (0.312, 1.020)	0.805 (0.398, 1.630)	0.809 (0.392, 1.668)
CCI score	**1.206 (1.045, 1.391)**	**1.295 (1.095, 1.531)**	**1.182 (1.004, 1.392)**	1.208 (0.997, 1.463)
Diabetes	0.904 (0.526, 1.555)	0.785 (0.325, 1.898)	0.903 (0.471, 1.730)	0.562 (0.219, 1.442)
Triglyceride (mmol/L)	1.281 (0.980, 1.673)	1.311 (0.981, 1.752)	0.777 (0.517, 1.166)	0.808 (0.535, 1.220)
Cholesterol (mmol/L)	0.975 (0.839, 1.132)	0.903 (0.745, 1.094)	0.958 (0.795, 1.154)	0.961 (0.790, 1.169)
Hemoglobin (g/L)	0.993 (0.983, 1.004)	0.990 (0.978, 1.003)	1.000 (0.998, 1.013)	1.006 (0.992, 1.020)
Plasma albumin (g/L)	0.979 (0.936, 1.023)	0.990 (0.943, 1.040)	0.968 (0.916, 1.023)	0.963 (0.907, 1.024)

HR: hazard ratio; 95% CI: 95% confidence interval; MACCE: main adverse cardiovascular and cerebrovascular events; TCC: tunneled cuffed catheter; CCI score: Charlson comorbidity score.

Bold values indicates significant statistical differences.

In this cohort, a total of 76 (49.4%) deaths occurred. A total of 50 (64.9%) TCC patients died. Fourteen patients died from main adverse cardiovascular and cerebrovascular events, sixteen died from infection, and twenty died from other etiologies or had an unknown cause of death. A total of 26 (33.8%) AVF patients died. Eleven patients died from main adverse cardiovascular and cerebrovascular events, nine died from infection, and six died from other etiologies or had an unknown cause of death. However, the causes of death were not significantly different between the two groups (*p* = 0.282, Table 4).

**Table 4.** Cause of death and hospitalization in TCC and AVF patients after matching.

Cause	Death		*p*	Hospitalization		*p*
	TCC (*n* = 50)	AVF (*n* = 26)		TCC (*n* = 168)	AVF (*n* = 62)	
Infection	16 (32%)	9 (34.6%)	0.282	50 (29.8%)	11 (17.7%)	0.09
MACCE	14 (28%)	11 (42.3%)		27 (16.1%)	16 (25.8%)	
Others or unknow	20 (40%)	6 (23.1%)		91 (54.2%)	35 (56.5%)	

Infection: includes all other infectious diseases except the upper respiratory tract infection; MACCE: main adverse cardiovascular and cerebrovascular events; TCC: tunneled cuffed catheter; AVF: arteriovenous fistulas.

#### 3.2.2. Main adverse cardiovascular and cerebrovascular events (MACCEs)

As shown in Table 2, the cumulative MACCE rates were 33.7 per 100 patient-years in the TCC group and 29.5 per 100 patient-years in the matched AVF group (*p* = 0.043). The number of MACCEs in the TCC group was as follows: 13 episodes of heart failure, 16 episodes of stroke, three cases of encephalorrhagia, four myocardial infarctions, and 20 other events. In the matched AVF group, the number of MACCEs was as follows: eight episodes of heart failure, three episodes of stroke, four myocardial infarctions, and eight other events.

The crude HR and adjusted HR of the occurrence of first MACCE in the TCC patients compared with the AVF patients were 1.814 (95% CI 0.983–3.349) and 2.062 (95% CI 1.081–3.932), respectively (Table 3). Multivariate Cox survival analyses indicated that TCC is a risk factor for MACCEs.

**Figure 3.**
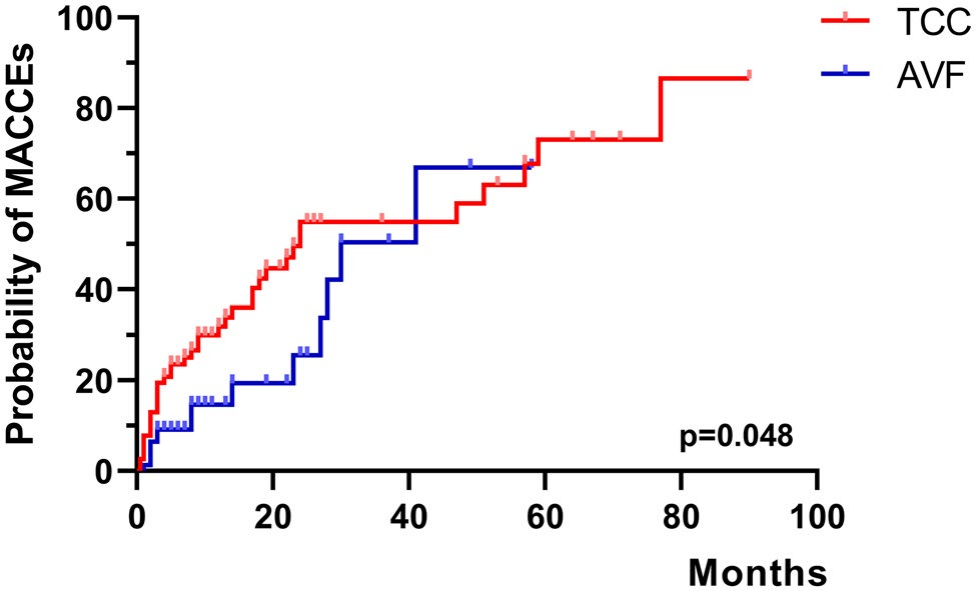
Risk of the occurrence of a first MACCE after PSM. MACCE: main adverse cardiovascular and cerebrovascular events; TCC: tunneled cuffed catheter; AVF: arteriovenous fistulas; PSM: propensity score matching.

The Kaplan–Meier curve also revealed that TCC was associated with a higher risk of the occurrence of a first MACCE (log-rank *p* = 0.048, Figure 3).

#### 3.2.3. Hospitalization

The cumulative hospitalization rates were 101.2 per 100 patient-years in the TCC group and 79.5 per 100 patient-years in the matched AVF group, and these differences were significant (*p* < 0.001) (Table 2). The reasons for hospitalization in the TCC group were as follows: 50 infections, 27 MACCEs, and 91 other events. In the matched AVF group, the reasons for hospitalization were as follows: 11 infections, 16 MACCEs, and 35 other events. However, the cause of hospitalization was not significantly different between the two groups (*p* = 0.09, Table 4).

#### 3.2.4. Infection

As shown in Table 2, 50 cumulative infection events occurred in the TCC group, and 11 cumulative infection events occurred in the AVF group during the observation period. Notably, 12 infection events were catheter-related infections in the TCC group. The cumulative infection rates were 30.1 per 100 patient-years in the TCC group and 14.1 per 100 patient-years in the matched AVF group. TCC is associated with a greater risk of infection (*p* < 0.001).

## 4. Discussion

In this retrospective observational cohort study, we compared the impacts of different vascular access types on all-cause mortality, MACCE rates, hospitalization rates, and infection rates in elderly HD patients aged ≥70 years who were treated between 1 January 2010 and 10 October 2023. Our study showed that, before PSM, mortality was significantly higher in patients with a TCC than in those with an AVF. However, in the propensity score-matched cohort, there was no difference in mortality between the TCC and AVF groups. We suggest that the disparity in mortality before matching may be attributed to differences in preexisting conditions, such as older age and higher CCI scores among the TCC patients. Furthermore, compared to AVF patients, patients with a TCC were associated with a significantly higher risk of MACCEs, and higher cumulative rates of hospitalization and infection as well.

The vascular access required for hemodialysis treatment is ideally satisfied with both high-flow and low-resistance. AVF is considered the first choice for vascular access. However, over the past decade, the traditional ‘fistula first’ concept has changed to the ‘patient first’ concept, which reflects the greater clinical emphasis on individual strategies while treating patients [3]. Currently, few national and international guidelines provide recommendations regarding which vascular access is best for elderly dialysis patients. Some studies suggested that AVF as initial access for long-term dialysis therapy was associated with the best survival [15], while others indicate no significant survival benefit among elderly HD patients with different access types (AVF, CVC, or conservative management) over a two-year period [16].

Our study initially revealed that there are better survival rates in the AVF group compared to the TCC group before matching. However, this difference disappeared after PSM, suggesting that confounding factors, such as older age and higher CCI scores in the TCC group may have influenced the initial results. The high prevalence of comorbidities in elderly patients, especially diabetes mellitus, peripheral vascular disease, and cardiovascular disease, can complicate VA creation [17]. Considering these factors, we propose that there may be no significant survival difference between TCC and AVF for elderly HD patients.

Our study demonstrated a higher cumulative hospitalization rate in the TCC group compared to the AVF group. Analyzing the reasons for hospitalization within our matched cohort revealed that TCC patients experienced higher rates of infection-related hospitalizations (29.8%) compared to AVF patients (17.7%). Conversely, AVF patients had a higher proportion of hospitalizations due to MACCEs (25.8%) compared to TCC patients (16.1%). These findings are consistent with existing research demonstrating significantly increased hospitalizations due to catheter-related bacteremia in patients using CVCs compared to AVFs [18]. Furthermore, the study suggests that catheter-based dialysis incurs substantially higher annual costs for access management [18]. Therefore, considering both infection rates and potential economic burden, our findings suggest that AVFs might be preferable to TCCs for elderly HD patients, potentially contributing to reduced hospitalizations and lower healthcare costs.

Our study revealed a higher incidence of heart failure among AVF patients (34.8%) compared to the TCC group (23.2%), which is consistent with existing literature linking long-term AVF placement with risks like left ventricular hypertrophy (LVH), congestive heart failure (CHF), myocardial ischemia, and venous stenosis [19]. After the formation of an AVF, the cardiac load increases, and there is a 10–25% increase in cardiac output (CO) [20]. This physiological adaptation occurs through a reduction in peripheral resistance, increased sympathetic activity, and higher stroke volume and heart rate [21]. Consequently, AVF patients may be more susceptible to heart failure, a finding consistent with previous research [22].

Our study identified a higher cumulative incidence of MACCEs in the TCC group compared to the AVF group, which may be due to the following reasons. First, in terms of cardiac contractile function and vascular conditions, we were unable to fully match the patients because of the difficulty of data collection. We consider that patients with a TCC have inherent poor vascular conditions, potentially characterized by narrow veins or severe arteriosclerosis, might have contributed to the observed differences. Second, we suspect that dialysis access recirculation and inadequate dialysis increase volume, decrease toxin clearance, enhance sympathetic activity and action in the cardiovascular system [23], and ultimately cause cardiovascular events. Unfortunately, because of the different follow-up times between the two groups, it is difficult to retroactively compare dialysis adequacy. Third, despite we performed propensity score matching, there are some potential factors that may still not be fully matched, so whether these factors affected the final outcome cannot be determined.

Our study showed a significantly higher cumulative number of infections in the TCC group compared to the AVF group, consistent with previous research reporting higher infection risks with CVC use [17,24,25]. While some studies suggest decreasing catheter-related bloodstream infection risks in elderly HD patients is due to reduced physical activity and external stresses on the catheter [26], the overall risk of infection of patients with a CVC remains a concern due to the potential entry of skin bacteria. Unfortunately, the lack of data collection on inflammation-related indicators limits our ability to fully evaluate systemic inflammatory conditions and their potential impact on patient outcomes. Notably, as previous research suggests, an episode of either bacteremia or hospitalized septicemia was a harbinger of future cardiovascular events and especially death [24]. Our findings reveal a concerning situation in TCC patients, demonstrating higher rates of both infections and MACCEs. Further research is necessary to elucidate the relationships among infections, MACCEs, and mortality in this population.

While several previous studies revealed that the use of a CVC was a risk factor associated with death [8,15,27], others have found this association non-significant after adjusting for confounding factors [28]. Our multivariate analysis similarly identified the use of TCCs was not a risk factor for death. A large-scale cohort study of elderly US patients proposes that the actual mortality attributed to the catheter itself is much lower than previously considered and may be explained in large part by differences in patient factors [29]. If patients treated with catheters are simply sicker, the majority of deaths could be due to comorbidities; therefore, these deaths are unrelated to complications of vascular access [30]. This challenges the claim of direct CVC-related mortality, arguing that complications from vascular access play a minor role [31]. Thus, it seems that TCC is not a risk factor directly affecting death, but instead due to differences in the health conditions of patients or may also be due to other complex and unmeasured confounding factors.

Several limitations of this study should be acknowledged. The main limitation is that this was not a randomized study but rather a retrospective cohort study. Despite the matching and adjustment for several confounding factors, residual confounding cannot be excluded. Therefore, this study may not be completely free of bias due to confounding factors. This was a single-center study, and we had access to a limited sample size. We failed to use 1:2 propensity score matching, which is more representative of the data. Additionally, the care and follow-up of patients during dialysis might affect the results. Second, we did not analyze outcomes for patients who experienced fistula failure or required a change in vascular access type during the study. This limits our understanding of how those events may impact mortality risk. Third, TCC placement is often performed in patients with multiple comorbidities and contraindications to surgery. There could be selection bias because healthier patients may be more likely to have AVF placement. Fourth, there were numerous patients whose cause of death was unknown. This potentially could confound our findings, especially with regard to the number of infection-related deaths.

## 5. Conclusion

In conclusion, our study demonstrated that the use of a TCC *vs.* an AVF did not significantly different regarding the mortality of HD patients ≥70 years old. Importantly, AVF patients were at a lower risk of the MACCEs, hospitalization, and infection compared to TCC patients. Thus, in terms of MACCEs, hospitalization, and infection, we recommend that AVF placement is superior to TCC placement for elderly patients ≥70 years old. However, the selection of vascular access type for elderly HD patients remains complex and must be individualized. Factors, such as life expectancy, comorbidities, vascular condition, and patient preferences should always guide decision-making.

## Data Availability

The datasets used and/or analyzed during the current study are available from the first author on reasonable request.
